# Does Pizza Consumption Favor an Improved Disease Activity in Rheumatoid Arthritis?

**DOI:** 10.3390/nu15153449

**Published:** 2023-08-04

**Authors:** Roberta De Vito, Maria Parpinel, Michela Carola Speciani, Federica Fiori, Rachele Bianco, Roberto Caporali, Francesca Ingegnoli, Isabella Scotti, Tommaso Schioppo, Tania Ubiali, Maurizio Cutolo, Giuseppe Grosso, Monica Ferraroni, Valeria Edefonti

**Affiliations:** 1 Department of Biostatistics, Data Science Initiative, Center for Computational Molecular Biology, Brown University, 121 South Main Street and 164 Angell Street, Providence, RI 02912, USA; roberta_devito@brown.edu; 2 Department of Medicine, University of Udine, Via Colugna 50, 33100 Udine, Italy; maria.parpinel@uniud.it (M.P.); federica.fiori@uniud.it (F.F.); bianco.rachele@spes.uniud.it (R.B.); 3 Branch of Medical Statistics, Biometry, and Epidemiology “G. A. Maccacaro”, Department of Clinical Sciences and Community Health, Università degli Studi di Milano, Via Celoria 22, 20133 Milan, Italy; michela.speciani@unimi.it (M.C.S.); monica.ferraroni@unimi.it (M.F.); 4 Rheumatology Clinic, ASST Gaetano Pini, Department of Clinical Sciences and Community Health, Research Center for Adult and Pediatric Rheumatic Diseases, Università degli Studi di Milano, Piazza A. Ferrari 1, 20122 Milan, Italy; roberto.caporali@unimi.it; 5 Rheumatology Clinic, ASST Gaetano Pini, Piazza A. Ferrari 1, 20122 Milan, Italy; isabella.scotti@asst-pini-cto.it; 6 Medicina Generale II, Ospedale San Paolo, ASST Santi Paolo Carlo, Via Antonio di Rudinì 8, 20142 Milan, Italy; tommaso.schioppo@asst-santipaolocarlo.it; 7 UO Reumatologia, ASST Papa Giovanni XXIII, Piazza OMS—Organizzazione Mondiale della Sanità 1, 24127 Bergamo, Italy; tubiali@asst-pg23.it; 8 Laboratory of Experimental Rheumatology and Academic Division of Rheumatology, Department of Internal Medicine, University of Genova—IRCCS San Martino Polyclinic Hospital, Viale Benedetto XV 6, 16132 Genova, Italy; mcutolo@unige.it; 9 Department of Biomedical and Biotechnological Sciences, Center for Human Nutrition and Mediterranean Foods (NUTREA), University of Catania, Via S. Sofia 97, 95123 Catania, Italy; giuseppe.grosso@unict.it; 10 Fondazione IRCCS Ca’ Granda Ospedale Maggiore Policlinico, Via Sforza 35, 20122 Milan, Italy

**Keywords:** cross-sectional study, DAS28, disease activity, Italy, pizza, mozzarella cheese, olive oil, refined grains, rheumatoid arthritis, SDAI

## Abstract

To our knowledge, no studies so far have investigated the role of pizza and its ingredients in modulating disease activity in rheumatoid arthritis (RA). We assessed this question via a recent cross-sectional study including 365 participants from Italy, the birthplace of pizza. Multiple robust linear and logistic regression models were fitted with the tertile consumption categories of each available pizza-related food item/group (i.e., pizza, refined grains, mozzarella cheese, and olive oil) as independent variables, and each available RA activity measure (i.e., the Disease Activity Score on 28 joints with C-reactive protein (DAS28-CRP), and the Simplified Disease Activity Index (SDAI)) as the dependent variable. Stratified analyses were carried out according to the disease severity or duration. Participants eating half a pizza >1 time/week (vs. ≤2 times/month) reported beneficial effects on disease activity, with the significant reductions of ~70% (overall analysis), and 80% (the more severe stratum), and the significant beta coefficients of −0.70 for the DAS28-CRP, and −3.6 for the SDAI (overall analysis) and of −1.10 and −5.30 (in long-standing and more severe RA, respectively). Among the pizza-related food items/groups, mozzarella cheese and olive oil showed beneficial effects, especially in the more severe stratum. Future cohort studies are needed to confirm this beneficial effect of pizza and related food items/groups on RA disease activity.

## 1. Introduction

Rheumatoid arthritis (RA) is a chronic inflammatory disease involving multiple joints, with a multifactorial and autoimmune pathogenesis [1]. The worldwide RA prevalence in 1986–2014 ranged from 0 to 2.70% (mean prevalence: 0.56%) [2]. In Italy, the RA prevalence in 2011 was reported to be 0.41%, for active and in-remission disease together (0.32% for active RA, and 0.09% for RA in remission, respectively) [3]. Although the RA prevalence in Italy was stable from the 1960s to the 1990s [4], a slight increase has been reported in more recent years [3,4], in line with the increase in age-standardized prevalence and incidence reported in Europe from 1990 to 2019, according to the most recent Global Burden of Disease data [5]. Given the significant effects of RA on patients’ physical function, quality of life, and emotional state [6], and the associated co-morbidities, RA development and treatment represent a major public health issue [5].

The clinical evolution of the disease has substantially improved over the years, due to advances in early diagnosis and treatment, with a particular emphasis on the role of modern biological disease-modifying anti-rheumatic drugs (DMARDs), and their use in the early stages of the disease, to prevent long-term complications [7,8]. While composite measures of disease activity (e.g., the Disease Activity Score based on 28 joints (DAS28), and the Simplified Disease Activity Index (SDAI)) are suggested [9,10], and currently used in clinical practice, a non-negligible proportion of patients still do not reach the goal of a sustained remission or, at least, of a low disease-activity state [11,12]. A positivity to autoantibodies, such as rheumatoid factor (RF) and/or anti-citrullinated protein antibodies (ACPA), a very high disease activity, and early joint damage are considered unfavorable prognostic factors [13,14]; the disease duration can also impact the response to treatment and the disease activity [15].

Both RA patients and clinicians [16] are continuously in search of novel solutions to integrate pharmacological therapy and alleviate the disease burden. Together with other environmental exposure factors [17], diet constitutes a suitable candidate, as its impact on systemic inflammation, oxidation, and the gut microbiota could ease disease activity [18,19,20]. While most observational studies concerning diet and RA are focused on the disease onset, the disease activity is generally investigated using short-term dietary interventions [20]. So far, these results have been either challenging to generalize, due to heterogeneity in several aspects of the design, or outdated treatment strategies, or they have been affected by flaws in the study design (e.g., a small sample size) and/or implementation [20,21]. Therefore, even where the evidence of a potential favorable effect on RA disease activity is stronger (i.e., the Mediterranean diet, oily fish, and omega-3 polyunsaturated fatty acids), additional research, based on longer, larger, and better-designed studies is still required [20,22,23]. In addition, more effective therapies might require a paradigm shift in the study design of RA activity investigations, from the short-term, likely temporary, effects of dietary interventions in trials on selected RA populations, to the long-term, likely modest but persistent, effects of regular dietary habits in observational studies on free-living RA patients [24,25,26]. Achieving a modest “real-life” benefit over time may be more critical in the long run than chasing even stronger “intervention-oriented” benefits that may disappear a few months after the intervention ends [27]. Within this perspective, inexpensive, palatable, and easily accessible foods that may not even require preparation are the first target to be investigated, especially for those patients who experience difficulties in managing everyday activities such as cooking [27]. Pizza meets all of these requirements.

Following the Mediterranean diet’s recognition in 2013, in 2017 the United Nations Educational, Scientific and Cultural Organization (UNESCO) inscribed the “art of Neapolitan pizzaiuolo (pizza maker)” on the Representative List of the Intangible Cultural Heritage of Humanity; Neapolitan pizza (Verace Pizza Napoletana) has been certified as Traditional Specialty Guaranteed (TSG) since 2010, and protected with regulatory and disciplinary action since 1984 [28,29]. Together with World Pizza Day, these recognitions testify that pizza, a cornerstone of “Made in Italy”, is now a universal food, loved and consumed all over the world. The top worldwide consumer countries include the US (13 kilos per capita per year), Italy (7.8 kilos per capita per year), and some other European countries, with a much lower yearly consumption of 3–4 kilos per capita [30]. Despite the incredibly high diffusion of pizza, studies on its role, as a single food item, on health are scanty, and are essentially limited to cardiovascular disease and cancer, investigated in Italy and the US. Within a network of Italian hospital-based case–control studies, a protective effect was observed on acute myocardial infarction and cancer of the oral cavity/pharynx, esophagus, and colon, but not on cancer of the larynx, rectum, breast, ovary, or prostate [31,32,33,34]; another population-based case–control study from southern Italy provided nonsignificant findings for colon and rectal cancers together [35]. While assessing the effect of lycopene food sources or tomato-based products, a few US studies investigated the possible role of pizza on cardiovascular disease and related biomarkers [36,37], or on prostate cancer [38], but the protective effect identified did not reach statistical significance. Although common mechanisms of inflammation and oxidation can be hypothesized across chronic diseases, to our knowledge, no studies so far have targeted the potential role of pizza consumption in modulating inflammation, and therefore reducing the disease activity, in RA. Putative anti-inflammatory foods, such as pizza, may play a direct as well as an indirect role, through the management of well-known RA-related co-morbidities, including obesity, cardiovascular disease, and diabetes [20].

Within Italian tradition, pizza is a stand-alone meal, usually made from fresh, high-quality ingredients, including mozzarella cheese (from cow milk, called “fior di latte”, or from buffalo milk, called “mozzarella di Bufala”, both far from pre-shredded mixed cheese), tomato sauce, and/or “pomodorini” (cherry tomatoes)—the thicker flesh and fewer seeds of which provide a sweeter flavor and a less acidic bite—and olive oil (mostly extra virgin olive oil), all over a lean dough (generally made with type 00 wheat flour, water, salt, and yeast) [28,29]. Pizza’s nutrient composition generally balances carbohydrates, proteins, and fats well [39]. Pizza is highly consumed across the different Italian regions, at home (58%) (delivered from pizzerias in 29% of cases), or in pizzerias (42%) [40,41]; it has also been reported that 44% of Italians make pizza at home [30]. Outside Italy, pizza generally belongs to a “Western-like” dietary pattern, along with other junk foods, including burgers and French fries [42,43,44,45,46]; this generally differs from Italian dietary patterns, where pizza has also been found to feature highly on an “Eggs and Sweets” pattern [47,48,49,50,51,52,53,54], including eggs and dairy products, and on a “Prudent” pattern, including vegetables, legumes, potatoes, and soup [55,56], reflecting a much more varied consumption of pizza in Italy, compared to other countries. As an example of the pizza market outside of Italy, of the over 75,000 pizzerias in the US, 29.2% are chain restaurants—with Pizza Hut being the largest global chain—and frozen pizza accounts for $21.5 billion of the $41.5 billion pizza industry [57]. These differences might suggest a need to separately investigate the role of pizza in Italy and outside of Italy.

In a recent cross-sectional analysis on RA patients from Italy, we aimed to investigate whether a higher consumption of pizza, the most famous example of an inexpensive, palatable, and easily accessible Italian food, might contribute to the improved management of RA disease activity in free-living individuals. We additionally aimed to investigate the role of the available pizza-related food items/groups (i.e., refined grains, mozzarella cheese, and olive oil), to assess whether these food items/groups, and which ones among them (consumed as stand-alone foods in pre-specified reference portions and periods) might be responsible for any effect observed of pizza consumption on RA disease activity.

## 2. Materials and Methods

### 2.1. Design and Participants

The current analysis was based on a previously published [21,27] cross-sectional study on dietary habits and disease activity in RA patients conducted in Milan, Italy, at the in- and out-patient rheumatology clinic, Gaetano Pini Hospital, from January 2018 to December 2019 (granted ethical approval: 751_2017bis, Comitato Etico Milano Area 2). The first publication, on the Mediterranean diet and RA disease activity [58], included the first 205 participants of the current investigation. Briefly, the enrolled RA patients, aged between 18 and 65 years, had a disease duration of 3 months minimum, and conformed to the classification criteria identified by the American College of Rheumatology (ACR) in 1987 [59], and/or the ACR/European League Against Rheumatism in 2010 [60].

### 2.2. Data Collection

**Figure 1:**
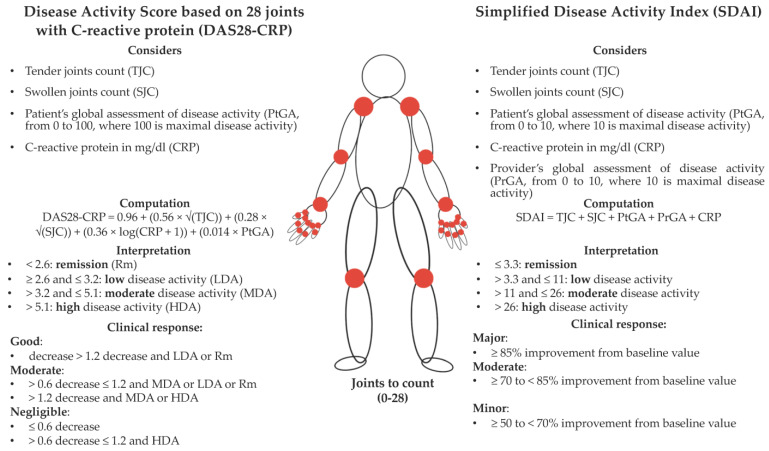
Composite measures of rheumatoid arthritis disease activity: the Disease Activity Score based on 28 joints with C-reactive protein (DAS28-CRP), and the Simplified Disease Activity Index (SDAI) in comparison. Log stands for natural (base e) logarithm; the DAS28-CRP measures, activity thresholds, and clinical response definition, are reported according to Fransen et al., 2009 [65]; the DAS28-CRP remission threshold is reported according to the American Rheumatism Association definition [66]; the SDAI measures, activity/remission thresholds, and clinical response definition, are reported according to Smolen et al., 2014 [67].

Details on the data collection procedures have been given elsewhere [21,27,58]. Briefly, centrally trained interviewers collected the participants’ information regarding sociodemographic characteristics, anthropometric factors, cigarette smoking, alcohol drinking, and a detailed medical history; the RA disease activity (measured using the DAS28 with C-reactive protein (CRP) (indicated as DAS28-CRP from here onwards) and the SDAI), the current RA treatment, the patient’s general health, the relevant laboratory parameters (i.e., the CRP and erythrocyte sedimentation rate), and the physician’s global assessment were also recorded (see Figure 1 for additional details). The participants filled in information on their usual intake of single items (foods and beverages) within a reproducible and valid [61] 110-item food frequency questionnaire (FFQ), by choosing among frequency categories mainly ranging from “never” to “4–5 times/day”. Suitable food composition tables (i.e., the Italian Research Center for Foods and Nutrition [62], and the US Department of Agriculture (USDA) National Nutrient Database for Standard Reference version 2011 [63], when needed) allowed the calculation of the individual intakes of the selected nutrients, and the total energy [64].

### 2.3. Selection of Participants and Variables

Among the 366 study participants, 39 subjects showed an extreme (i.e., <5th or >95th percentile) total energy intake, potentially pointing to unreliable dietary information in either direction (i.e., under-reporting or over-reporting); the FFQ information from these patients was carefully checked for the completeness of the items filled in, and compared with the patient’s weight, height, and how their disease likely impacted their daily living activities (e.g., the patient spending most of his/her time in a wheelchair). Specifically, only one of these participants (showing a total energy intake < 1000 kcal) was confirmed to have unreliably filled in the FFQ (i.e., missing items were detected in several sections of the FFQ), and thus was excluded. A total of 365 subjects were therefore included in our analyses.

The current analysis focused on the effect on RA disease activity of freely and ad libitum eating pizza and the available related food items/groups (according to the intake per day, with the frequency categories and standard portions in parentheses for the FFQ food items): (1) pizza (food item, nine frequency categories, from “never” to “4–5 times/day”, half a pizza), (2) refined grains (food group, better described below), (3) mozzarella cheese (food item, nine frequency categories, from “never” to “4–5 times/day”, 1 serving of mozzarella), and (4) olive oil (food item, four frequency categories, from “never” to “≥2 times/day”, 1 tablespoon). The refined-grains food group included the following food items (nine frequency categories, from “never” to “4–5 times/day”, with the single standard portions in parentheses): bread and bakery products (e.g., cookies, crackers) (100 g), pasta (80 g), rice (80 g), cornflakes or wheat cereal, (30 g), and corn (100 g).

### 2.4. Statistical Analysis

For each food item/group, the study participants were divided according to the tertiles of consumption calculated for the overall population.

We then adopted multiple regression models that considered the RA disease activity as the dependent variable, measured using either the DAS28-CRP or the SDAI, and the tertile-based categories of each food item/group as the main independent variable (expressed as the two highest categories of consumption, indicated as II or III in the following tables, vs. the lowest one, i.e., the reference category, indicated as I in the table footnotes).

The disease activity was expressed either in continuum or as a binary variable (i.e., the presence of a low, moderate, or high disease activity, vs. remission). Specifically, if the RA disease activity variable was assumed to be continuous, the regression models estimated the beta coefficient representing the mean increase/decrease in RA disease activity and its corresponding standard error (SE), according to the highest tertile-based consumption categories of each food item/group (vs. the lowest one). As the linear regression models’ adherence to the standard ordinary least-squares assumptions was violated, we opted for the robust regression model with an MM estimator [68]. Instead, if the RA disease activity variable was assumed to be binary, we adopted the logistic regression model, and estimated the odds ratios (ORs) of the RA disease activity (vs. remission), and its 95% confidence intervals (CIs), for each of the two highest consumption categories of each food item/group, vs. the lowest one.

In each model adopted in the analyses, potential confounding variables were considered, and adjustment was included for: age (≤55, >55 years old), sex (male, female), education level (primary school, middle school, high school, or university), total energy intake, body mass index (BMI of <18.5, 18.5–24, 25–29, ≥30 kg/m^2^), alcohol-drinking intensity (never a drinker, <1, 1–<2, ≥2 drinks/day, where 1 drink/day = 12 g of ethanol in the Italian population [69]), cigarette-smoking status (never, former, current), presence of any therapy (yes, no), conventional synthetic (cs)DMARDs (no, yes), biologic (b)DMARDs (no, yes), targeted synthetic (ts)DMARDs (no, yes), steroids (no, yes), disease duration (≤5, 5–≤10, 10–≤15, 15–≤25, >25 years), RF (negative, positive), and ACPA (negative, positive).

Moreover, we carried out stratified analyses according to the disease duration (≤15 years, >15 years) and disease severity (RF- and ACPA-negative, RF- and/or ACPA-positive) for any of the previously fitted models. The heterogeneity across strata was also assessed by adopting the likelihood ratio test. Additionally, two sensitivity analyses were performed on subjects (1) with normal blood pressure, or (2) not reporting either gastro-esophageal reflux or gastritis.

The statistical analyses were performed via the open-source statistical environment R [70], and its libraries “MASS” [71], “robustbase” [72], and “xlsx” [73].

## 3. Results

### 3.1. Study Population Characteristics: Most Females in Remission or with Low Disease Activity

Table S1 shows the sociodemographic characteristics, clinical features, and pharmacological therapy of the RA patients recruited into this study. The specific details were presented in a recent manuscript [27]. Briefly, the median age of the participants was 58.46 (interquartile range (IQR): 47.81–69.03) years, and 78.63% were female. The median disease activity was 2.21 (IQR: 1.61–3.02) when measured using the DAS28-CRP, and 6.30 (IQR: 3.01–11.81) when measured using the SDAI; according to the two indexes, the percentages of subjects in remission were equal to 62.19% and 29.59%, respectively. Rheumatoid factor and ACPA positivity were identified in 53.70% and 50.96% of the sample, respectively. The median disease duration was 12.81 (IQR: 8.08–20.72) years.

### 3.2. Summary Statistics of Consumption Frequencies of Pizza and Related Food Items/Group: Low Percentages of Non-Consumers, Differences in Summary Statistics between Overall Sample and Strata for All Investigated Variables, Except for Refined Grains

Table 1 presents the summary statistics of the consumption frequencies of the four food items/groups, as freely consumed by the RA patients considered in the current analysis. For each food item/group, the percentages of non-consumers were similar overall, and across strata, and they were generally very low, reaching ~12% for mozzarella cheese in the overall sample. The pizza consumption showed an overall median of 0.142, which corresponded to half a pizza (reference portion) once a week, with the third quartile still being equal to 0.142, and the first one being 0.032 (i.e., half a pizza 1 time/month). The same summary statistics were observed across the strata of the duration, and for the RF- and ACPA-negative stratum; on the other hand, participants experiencing a more severe RA form showed a lower median consumption of pizza, of 0.065 (i.e., half a pizza 2 times/month). For the mozzarella cheese food item, the overall median and the third quartile were equal to 0.142, which corresponded to one serving of mozzarella (reference portion) 1 time/week. The differences across strata concerned only the first quartile, which was higher when the RA severity or duration were lower, compared to the remaining strata. In detail, in the overall analysis and the remaining strata, the first quartile was equal to 0.032 (1 serving of mozzarella, 1 time/month) vs. 0.065 (1 serving of mozzarella, 2 times/month) in the RA duration ≤15 years, or the RF- and ACPA-negative strata. Therefore, the 25% of our sample with a less severe or not long-standing RA, ate mozzarella cheese more frequently. Differently to pizza and mozzarella cheese, the olive oil food item reached the highest median of 3—which corresponded to 1 tablespoon ≥2 times/day, and the maximum of the distribution—in the less severe or long-standing RA forms, compared to a median of 1 (i.e., 1 tablespoon, 1 time/day) in the overall analysis, and in the remaining strata. Finally, the refined grain food group presented an overall median of approximately two standard portions/day among pasta, rice, bread, cornflakes, and corn, with the first quartile equal to approximately 1 portion/day and the third quartile equal to approximately 3 portions/day. The summary statistics were similar to the overall analysis in the four strata under examination.

### 3.3. Beneficial Effect (the DAS28-CRP and SDAI), Mostly Significant, in the Highest Tertile Category of Pizza Consumption

Table 2 shows the ORs of RA disease activity, with their corresponding 95% CIs (upper panel), and the beta coefficients, with their corresponding standard errors (lower panel), by the tertile-based categories of consumption (II or III vs. I) of the four food items/groups under investigation. The estimates were obtained via logistic and robust regression models, including the single food item/group as the main exposure variable, and the confounders identified in the overall sample.

Participants in the highest consumption category of the pizza food item (i.e., eating the reference portion of half a pizza >1 time/week, III, vs. ≤2 times/month, I) generally reported beneficial effects on disease activity (three out of four fitted models). Specifically, in the logistic regression models, a significant beneficial effect was observed for the SDAI only, with protection around 70% (OR = 0.274, 95% CI: 0.079–0.952) for individuals eating the reference portion of half a pizza >1 time/week (i.e., III). In the robust regression models, the beneficial effect was evident for both the DAS28-CRP and SDAI, with the corresponding beta coefficients equal to −0.730 (SE: 0.350, *p*-value: 0.044) and −3.587 (SE: 1.537, *p*-value: 0.021) for those who ate the reference portion of half a pizza >1 time/week (i.e., III) for the DAS28-CRP and SDAI, respectively. Referring to the possible effects of pizza-related food items/groups, mozzarella cheese was the one that likely exerted the strongest beneficial effect on RA activity. Significance was observed only for the SDAI in the logistic regression model, where a ~70% risk reduction (OR: 0.321, 95% CI: 0.151–0.683), similar to the pizza food item, was evident among those in the third tertile category (i.e., eating the reference portion of one serving of mozzarella >1 time/week, III), vs. those in the first one (i.e., ≤2 times/month, I).

### 3.4. Strata by Disease Severity: Stronger Beneficial Effect (the DAS28-CRP and SDAI), Mostly Significant, in the Highest Tertile Category of Pizza Consumption, for the More Severe Stratum, Mirrored in Part by Mozzarella Cheese and Olive Oil

Table 3 displays the results of the stratified analysis on the more severe (i.e., RF- and/or ACPA-positive participants, left panel) and the less severe (i.e., RF- and ACPA-negative participants, right panel) RA variants for pizza and the related food items/groups. For the pizza food item, we consistently observed that the beneficial effect of the highest consumption categories was stronger in those who showed the more severe RA variant (RF and/or ACPA positivity), further corroborated by the presence of heterogeneity in the results across the disease severity strata (*p*-values < 0.05 for all four models). In detail, an 80–85% reduction in risk was present for both the DAS28-CRP and SDAI in the logistic regression models for those in the third tertile (i.e., those who ate the reference portion of half a pizza >1 time/week, III) who experienced the more severe RA variants (for the DAS28-CRP, OR: 0.195, 95% CI: 0.039–0.969, for the SDAI, OR: 0.161, 95% CI: 0.032–0.825). Similarly, in the robust regression models, participants with a more severe disease showed generally stronger effects, with the strongest benefit at the third tertile observed for the SDAI (beta: −5.279, SE: 2.053, *p*-value: 0.012). Concerning the possible effect of the pizza-related food items/groups, participants in the more severe strata, and in the highest-tertile consumption category of mozzarella cheese, experienced a stronger beneficial effect in three vs. one (out of the four) fitted models in the overall analysis. Specifically, a ~60% (vs. ~50%, non-significant, in the overall analysis) and ~80% (vs. ~70%, significant, in the overall analysis) risk reduction was observed for the DAS28-CRP (OR: 0.369, 95% CI: 0.141–0.968) and the SDAI (OR: 0.200, 95% CI: 0.068–0.586), respectively, in the logistic regression models for mozzarella cheese, although in the absence of heterogeneity between the strata. In the robust regression models, the beta coefficients for mozzarella cheese were stronger than in the overall analysis and reached significance when the DAS28-CRP was considered (beta: −0.573, SE: 0.230, *p*-value: 0.013), despite the *p*-value for heterogeneity across the strata exceeding 0.05 (i.e., 0.164). Additionally, olive oil revealed a beneficial effect in those with a more severe RA: when participants consumed >1 tablespoon/day (II) (vs. ≤1 tablespoon/day, I), the beta coefficient for the SDAI was −2.094 (SE: 0.962, *p*-value: 0.034), vs. −0.865 (SE: 0.654, *p*-value: 0.190) in the overall analysis, with a significant *p*-value for the heterogeneity across the strata (i.e., *p*-value< 0.001); the beta coefficient for the DAS28-CRP was equal to −0.371 (SE: 0.161, *p*-value = 0.023), vs. −0.201 (SE: 0.146, *p*-value: 0.171) in the overall analysis, in the absence of heterogeneity between the strata (*p*-value: 0.126).

### 3.5. Strata by Disease Duration: Beneficial Effect (DAS28-CRP and SDAI) in the Highest-Tertile Category of Consumption of Pizza and Mozzarella Cheese, for the Longer-Duration Stratum, within a General Framework of Non-Significant Findings

Table 4 displays the results of the stratified analysis on longer (i.e., >15 years, left panel) and shorter (i.e., ≤15 years, right panel) disease durations for the considered food items/groups. A beneficial effect of pizza, mozzarella cheese, and olive oil on RA disease activity was found among participants in the longer disease stratum, although few models reached statistical significance. Specifically, in robust linear regression models, the mean DAS28-CRP decreased when participants consumed the reference portion (i.e., either half a pizza or one serving of mozzarella) >1 time/week, vs. ≤2 times/month (for pizza, beta: −1.120, SE: 0.479, *p*-value: 0.018 for III vs. I, p-heterogeneity: 0.016; for mozzarella cheese, beta: −0.651, SE: 0.285, *p*-value: 0.023 for III vs. I, p-heterogeneity: 0.038). In addition, mozzarella cheese was associated with a significant ~80% (vs. a borderline significant ~50% in the overall analysis, and a significant ~60% in the more severe stratum) risk reduction (OR: 0.178, 95% CI: 0.049–0.650), when consumed >1 time/week (i.e., III) by participants with long-standing RA (*p*-value for heterogeneity across strata equal to 0.011). Finally, when considering the third vs. first tertile categories of consumption (III vs. I), the refined grain food group was not significantly related to the RA disease activity in the overall or stratified analyses.

Sensitivity analyses targeted study subjects with either normal blood pressure, or who had not reported suffering from gastritis or gastroesophageal reflux. The results were generally in line with those from the complete-case analysis, although based on smaller sample sizes. Details are provided in the Supplementary Materials, Results, text.

## 4. Discussion

In this cross-sectional study on RA patients, participants in the higher consumption categories of the pizza food item (i.e., those freely eating the reference portion of half a pizza >1 time/week vs. ≤2 times/month) reported beneficial effects on the disease activity, with statistical significance observed in three out of four models fitted on the overall sample, in three out of four models fitted on the more severe stratum, and in one of the three models fitted on the long-standing RA stratum. When significant, the reductions were around 70% in the overall analysis, and reached 80% in the more severe stratum, both from logistic regression models; from linear models, the significant beta coefficients were in the order of −0.70 for the DAS28-CRP and −3.6 for the SDAI and increased by 1.5 (reaching about −1.10 and −5.30) in the two strata of long-standing and more severe RA, respectively. Among the pizza-related food items/groups, eating the reference portion of one serving of mozzarella >1 time/week (vs. ≤2 times/month) was likely to exert the most beneficial effects, with statistical significance observed in one out of four models fitted on the overall sample (70% protection, similar to pizza), in three out of four models fitted on the more severe stratum (60 and 80% protection in logistic regression, and −0.57 for the DAS28-CRP in robust regression), and in two of the four models fitted on the long-standing RA stratum (>80% protection in logistic regression, and −0.65 for the DAS28-CRP in robust regression). Finally, olive oil generally exerted a beneficial effect on the more severe stratum, with statistical significance observed in two out of the four fitted models (−0.37 and −2.09 for the DAS28-CRP and SDAI in robust regressions, respectively).

Pizza is a natural candidate when looking for an easily accessible, tasty, and affordable food all over the world. The specificities of pizza consumption in Italy further favor RA patients, for two additional reasons:

(1) Pizza is generally intended as a stand-alone single-item meal in Italy, where typically no small, medium, or large size variants of pizza are served; RA patients can buy one pizza and, in this way, easily solve lunch/dinner cooking issues.
(2) Despite their high-quality ingredients, 96% of Italian takeout or restaurant pizzas currently cost between 5 and 10 euros [74]; eating a pizza once a week is therefore widely affordable, especially when compared to other foods, such as oily fish, walnuts, and seeds, or dietary supplements, such as omega-3 fatty acids, which are typically suggested to integrate the diet of RA patients [20].

However, to our knowledge, no studies so far have investigated the role of pizza consumption in RA management, in Italy or in the rest of the world. On the other hand, as a cheap, mass-produced food, pizza may also be assumed to contain poor-quality ingredients [75]; in the effort to enhance flavor, palatability and, ultimately, consumer acceptance, pizza recipes may include higher proportions of cheese and salt than is recommended [75,76]. This does not necessarily apply to Italian pizza, for the following reasons:

(1) Italian pizza’s composition generally balances carbohydrates, proteins, and fats well [39]. Should further evidence support our claim of improved RA activity with increased pizza consumption, nutritionists may suggest that RA patients eat pizza as a stand-alone meal more than once a week, while taking care not to exceed the suggested dietary target for sodium intake [69], which is easily reached through pizza consumption [39,76].
(2) Italian pizza is easier to digest, because high-quality tomato sauce is generally used [77], and the pizza is cooked at scorching temperatures [78]. This is a point in favor of pizza consumption in RA patients, who show an increased prevalence of gastro-esophageal reflux [79].
(3) In Italian pizza, not only does the emulsion of oil with tomato sauce before the cooking phase contribute to the uniform cooking of the ingredients [28], but it generally enhances the pizza’s antioxidant potential, including the content of phenolic compounds and lycopene, the Trolox Equivalent Antioxidant Capacity, and the bio-accessibility of phenolic compounds and lycopene [77]. Concerning oil, the higher-quality oil (e.g., olive oil or even extra-virgin olive oil), which is mandatory in Neapolitan Pizza TSG [28,29], provides greater resistance to heating-related lipid oxidation due to the higher presence of bioactive compounds such as polyphenols [80,81,82,83,84]. Concerning tomato sauce, which is mandatory in Neapolitan Pizza TSG [28,29], the formation of micelles with oil lipids facilitates lycopene release, following the initial heating-related partial breaking of the cell-wall membrane [77,85], thus increasing lycopene bio-accessibility. Finally, extra-virgin olive oil offers a suitable environment for the isomerization of lycopene, with the formed Z-isomers outperforming E-isomers in absorption, transport flexibility, and antioxidant capacity [77,86].
(4) Jointly with its antioxidant potential [77], the anti-inflammatory potential of Italian pizza may be another important mechanism of action. An increasing amount of evidence points to a beneficial effect, if any, of dairy products and dairy proteins on the biomarkers of inflammation [87,88], meaning that mozzarella cheese may exert intrinsic anti-inflammatory activity. In addition, cheese may counteract the glycemic response from the dough-related carbohydrate load [89], thanks to the presence of high-biological-value dairy proteins [69]. As an example, the Neapolitan pizza TSG requires 80 to 100 g of mozzarella on each pizza base, which corresponds to ~15–19 g of high-biological-value dairy proteins per pizza [28,29,39]. This may be particularly important when mozzarella on pizza is preferred to pro-inflammatory protein sources, such as processed meat [90], thus also exerting its anti-inflammatory effect through substitution.
(5) Finally, pizza might be thought a general indicator of a healthy, varied Italian diet, a variant of the Mediterranean diet that includes a higher consumption of pasta compared to other countries on the Mediterranean basin [34]. Although pizza itself does not, each of the single food items/groups investigated belongs to the definition of the Mediterranean diet [34]. The well-known anti-inflammatory and antioxidant properties of which have the potential to modulate inflammatory pathways in RA, and to benefit the gut microbiota [91,92,93]. These properties may be one of the reasons why, among European countries, Italy is in the top ten in terms of life expectancy at birth in 2021 (82.7 years) [94]. However, when adherence to the Mediterranean diet itself [95] was considered in relation to the DAS28-CRP or SDAI in a subset of the current sample, the results were materially null [58]. This similarly happened in two other observational studies from Greece [96] and Japan [97], assessing a potential association between dietary habits and disease activity although, in the latter [97], single components of the Mediterranean diet exerted some beneficial effect. Whether this was due to the difficulty in capturing the expected small effects of dietary habits in observational studies on RA activity, methodological issues in the study design/implementation, or other reasons, is still a matter of debate [20]. However, while we are observing a shift toward more motivated patients, engaged in the self-management of their disease, large and well-conducted cohort studies that consider reproducible and valid tools for diet assessment, internationally recognized measures of RA activity, and an appropriately wide set of confounding factors are urgently needed to draw firm conclusions on the role of diet in RA management [20]. This evidence will be integrated with that from dietary intervention trials in RA (see Philippou et al., 2021 [98], for an updated systematic review on the topic), where larger and longer-duration intervention studies are still needed, although the evidence of a beneficial effect of an anti-inflammatory Mediterranean diet in RA management seems stronger [20].

The described study has strengths and limitations. Among the strengths, we mention the use of a reproducible and valid FFQ for capturing dietary habits [61], and of two well-known composite measures of disease activity [58] for capturing the outcome variables, according to different criteria. In addition, we provided two parallel analyses, with logistic and robust linear regression models, to allow the results’ comparison with the thresholds of minimal clinically important improvements in the DAS28-CRP and SDAI through robust linear regressions (see Figure 1 for details), while closely following the distribution of RA patients with in-remission/active disease in our sample through logistic regressions [27]. In the absence of any evidence on this topic, the consistent findings we obtained across the different analysis approaches support the validity of our results. Among the several investigated confounding factors, we also adjusted all the regression models according to the total energy intake, to account for the differences in physical activity, body size, and metabolism, which impacted the total food consumption [64]. Finally, as pizza is typically consumed within the Italian culinary tradition, all the different frequencies of consumption are more likely to be well represented in Italy, and this provides the necessary capacity to investigate the role of pizza consumption in chronic diseases [33]. Among the study limitations, firstly, we mention our cross-sectional study design, which did not allow us to derive firm conclusions on the role of diet in RA disease activity. Secondly, although the FFQ reference period was small, it might be that RA patients adapted their diet during that period to follow the disease course; in this case, we cannot assess any “usual” dietary behavior—as typically happens with an FFQ—because no “usual” diet can be tracked in RA patients’ real life. Thirdly, we could not materially assess the effect of tomato sauce, a pizza ingredient that is as important as mozzarella cheese (20%), and more important than olive oil (4%), in Italian pizza [31], because our FFQ did not query tomato sauce consumption. Similarly, we could not compensate for this lack of information by using lycopene—which tomato sauce is rich in [39,63]—because the adopted food composition table did not provide estimates for this micronutrient [62]. Fourthly, Italian pizza is prepared following different regional recipes. Not only might there be different toppings, but pizza might also be served and consumed baked or fried, in slices of different shapes, as a “Calzone” (i.e., a portable pizza that can be eaten while walking or standing), or as a “Pizza al Taglio” (i.e., a slice cut from a bigger pizza, and sold by weight). Our analysis was not able to capture either these variants, or the contribution of toppings to the total energy intake. However, the lack of collected information on toppings is compensated by Italians’ clear preference (45% [40]) for pizza Margherita (San Marzano tomatoes, mozzarella cheese, fresh basil, and a drizzle of extra-virgin olive oil). Fifthly, although we accounted for several confounding factors in the fitted regression models, there might be additional unmeasured or measured-with-error confounders (e.g., depression) that might modify the relationship between diet and RA activity. In addition, while we collected over 350 RA patients from a single center, the stratified analyses conducted in the current application might have benefited from an even larger sample size. Finally, although our results shed light on an interesting research hypothesis, their generalizability is likely limited to those European countries that show similar RA prevalence and therapy protocols, and benefit from a similar culinary tradition and pizza ingredients’ availability.

## 5. Conclusions

To our knowledge, this is the first study investigating whether a higher consumption of pizza (and related food items/groups) could improve the composite, internationally recognized measures of RA disease activity. This study was conducted in Italy—the birthplace of pizza, and second-top consumer country of pizza worldwide—where access to the best pizza ingredients in their freshest state, and certified recipes provide the greatest likelihood of identifying the protective anti-inflammatory and antioxidant effects that pizza is believed to exert. In line with the expected results, participants consuming half a pizza >1 time/week (vs. ≤2 times/month) did report beneficial effects on disease activity, both in the overall analysis, and when the more severe RA forms were considered. These beneficial effects were likely driven by mozzarella cheese and, to a lesser extent, by olive oil, even though we were unable to assess the possible contribution of tomato sauce. These results require confirmation based on properly designed cohort studies that implement an assessment of diet with reproducible and valid tools, and employ internationally recognized measures of RA activity, to find the expected small dietary effects, and to adjust for the large set of confounders typical of RA. As our results are mostly based on patients with optimal disease control, the extent of the beneficial effect observed could be even greater if RA patients with active disease were primarily considered within these studies.

## Figures and Tables

**Table 1:** Summary statistics of the frequencies of consumption of the four investigated food items/groups in the overall population, and in the strata of disease severity and duration (reference portions shown in the footnote). The frequency of non-consumers is also shown for each food item/group. Italy 2018–2019.

Analyses		Total		Pizza	Refined Grains	Mozzarella Cheese	Olive Oil
**Overall**		*n* = 365	*n* (%) non-consumers	37 (10.14%)	5 (1.37%)	43 (11.78%)	17 (4.66%)
			median (I–III quartile)	0.142 (0.032–0.142)	1.785 (1.142–2.785)	0.142 (0.032–0.142)	1.000 (1.000–3.000)
			mean (SD)	0.107 (0.110)	2.030 (1.273)	0.159 (0.179)	1.868 (1.111)
**Severity**	**RF- and/or ACPA-positive**	*n* = 223	*n* (%) non-consumers	23 (10.31%)	1 (0.45%)	26 (11.66%)	11 (4.93%)
			median (I–III quartile)	0.065 (0.032–0.142)	1.785 (1.158–2.749)	0.142 (0.032–0.142)	1.000 (1.000–3.000)
			mean (SD)	0.103 (0.105)	1.954 (1.061)	0.160 (0.191)	1.791 (1.118)
	**RF- and ACPA-negative**	*n* = 142	*n* (%) non-consumers	14 (9.86%)	4 (2.82%)	17 (11.97%)	6 (4.23%)
			median (I–III quartile)	0.142 (0.032–0.142)	1.798 (1.141–3.000)	0.142 (0.065–0.142)	3.000 (1.000–3.000)
			mean (SD)	0.115 (0.117)	2.149 (1.546)	0.157 (0.161)	1.989 (1.094)
**Duration**	**Disease duration > 15 years**	*n* = 154	*n* (%) non-consumers	16 (10.39%)	3 (1.95%)	21 (13.64%)	5 (3.25%)
			median (I–III quartile)	0.142 (0.032–0.142)	1.714 (1.137–2.808)	0.142 (0.032–0.142)	3.000 (1.000–3.000)
			mean (SD)	0.111 (0.133)	2.001 (1.288)	0.152 (0.171)	1.977 (1.100)
	**Disease duration ≤ 15 years**	*n* = 211	*n* (%) non-consumers	21 (9.95%)	2 (0.95%)	22 (10.43%)	12 (5.69%)
			median (I–III quartile)	0.142 (0.032–0.142)	1.915 (1.190–2.714)	0.142 (0.065–0.142)	1.000 (1.000–3.000)
			mean (SD)	0.105 (0.089)	2.051 (1.265)	0.164 (0.185)	1.789 (1.116)

Frequency of consumption of each food item/group according to the reference portion (see below): Never = 0, 1/month = 0.032, 2/month = 0.065, 1/week = 0.142, 2–3/week = 0.357, 4–5/week = 0.643, 1/day = 1, 2–3/day = 2.5, 4–5/day = 4.5. Reference portion: **Pizza**: half a pizza; **Refined grains**: Bread: 100 g, Pasta or Rice: 80 g; Breakfast cereals: 30 g, and Corn: 100 g; **Mozzarella cheese**: 1 serving of mozzarella; **Olive oil**: 1 tablespoon. Abbreviations: ACPA, anti-citrullinated protein antibodies; *n*, number of subjects; RF, rheumatoid factor; SD, standard deviation.

**Table 2:** The odds ratios of rheumatoid arthritis disease activity, and the corresponding 95% confidence intervals (upper panel) and beta coefficients, representing an increase/decrease in the mean DAS28-CRP or SDAI, and the corresponding standard errors (lower panel), according to the highest tertile categories of consumption of the four investigated food items/groups: pizza, refined grains (without pizza), mozzarella cheese, and olive oil ^1^. Overall analysis. Italy 2018–2019.

Overall Analysis					
Logistic Regression					
Food Items/Group	Tertile Categories	Number of Subjects in Remission/Active RA	OR	95% CI	
**DAS28-CRP**					
Pizza	II (0.065; 0.142]	101/59	0.821	0.484	1.391
	III (0.142; 1]	14/3	0.252	0.062	1.028
Refined grains (without pizza)	II (0.142; 2.357]	66/47	1.900	1.002	3.602
	III (2.357; 9.675]	76/42	1.135	0.550	2.344
Mozzarella cheese	II (0.065; 0.142]	93/58	0.852	0.496	1.465
	III (0.142; 1]	46/18	0.490	0.236	1.018
Olive oil ^2^	II (1; 3]	112/60	0.729	0.443	1.201
**SDAI**					
Pizza	II (0.065; 0.142]	52/108	0.484	0.269	0.870
	III (0.142; 1]	7/10	0.274	0.079	0.952
Refined grains (without pizza)	II (0.142; 2.357]	32/81	0.641	0.313	1.315
	III (2.357; 9.675]	39/79	0.489	0.224	1.068
Mozzarella cheese	II (0.065; 0.142]	40/111	0.788	0.423	1.466
	III (0.142; 1]	27/37	0.321	0.151	0.683
Olive oil ^2^	II (1; 3]	49/123	0.993	0.575	1.715
**Robust linear regression**					
**Food items/group**	**Tertile categories**	**Number of subjects**	**Beta**	**SE**	***p*-value**
**DAS28-CRP**					
Pizza	II (0.065; 0.142]	116	−0.356	0.157	0.024
	III (0.142; 1]	12	−0.730	0.350	0.044
Refined grains (without pizza)	II (0.142; 2.357]	80	0.168	0.145	0.251
	III (2.357; 9.675]	73	−0.051	0.162	0.754
Mozzarella cheese	II (0.065; 0.142]	102	−0.045	0.159	0.782
	III (0.142; 1]	38	−0.362	0.213	0.087
Olive oil ^2^	II (1; 3]	118	−0.201	0.146	0.171
**SDAI**					
Pizza	II (0.065; 0.142]	160	−1.302	0.683	0.056
	III (0.142; 1]	17	−3.587	1.537	0.021
Refined grains (without pizza)	II (0.142; 2.357]	113	0.541	0.838	0.525
	III (2.357; 9.675]	118	−0.469	0.935	0.617
Mozzarella cheese	II (0.065; 0.142]	151	0.294	0.723	0.688
	III (0.142; 1]	64	−1.372	0.919	0.135
Olive oil ^2^	II (1; 3]	172	−0.865	0.654	0.190

^1^ Estimated from multiple logistic regression models, or robust linear regression models adjusted for age (≤55, >55 years old), sex, education (maximum level attained: primary school, middle school, high school, university), total energy intake, body mass index (BMI, <18.5, 18.5–24, 25–29, ≥30 kg/m^2^), alcohol-drinking intensity (never a drinker, <1, 1–<2, ≥2 drinks/day), cigarette-smoking status (never, former, current), presence of any therapy (yes, no), conventional synthetic (cs)DMARDs (disease-modifying anti-rheumatic drugs, no, yes), biologic (b)DMARDs (no, yes), targeted synthetic (ts)DMARDs (no, yes), steroids (no, yes), disease duration (≤5, 5–≤10, 10–≤15, 15–≤25, >25 years), rheumatoid factor (RF) (negative, positive), and anti-citrullinated protein antibodies (ACPAs) (negative, positive). The reference category, I, included the lowest consumers of each food item/group, i.e., participants consuming up to the first tertile value included. ^2^ For the olive oil food item, the third tertile category (III) was not available, because the second tertile value was equal to 3, which was also the maximum consumption attainable for olive oil. The frequency of consumption of each food item/group according to the reference portion (see below): Never = 0, 1/month = 0.032, 2/month = 0.065, 1/week = 0.142, 2–3/week = 0.357, 4–5/week = 0.643, 1/day = 1, 2–3/day = 2.5, 4–5/day = 4.5. Reference portion: **Pizza**: half a pizza; **Refined grains**: Bread: 100 g, Pasta or Rice: 80 g; Breakfast cereals: 30 g, and Corn: 100 g; **Mozzarella cheese**: 1 serving of mozzarella; **Olive oil**: 1 tablespoon. Abbreviations: CI, confidence interval; DAS28-CRP, Disease Activity Score based on 28 joints with C-reactive protein; OR, odds ratio; SDAI, Simplified Disease Activity Index; SE, standard error.

**Table 3:** The odds ratios of rheumatoid arthritis disease activity and the corresponding 95% confidence intervals (upper panel) and beta coefficients, representing an increase/decrease in the mean DAS28-CRP or SDAI, and the corresponding standard errors (lower panel), according to the highest tertile categories of consumption of the four investigated food items/groups: pizza, refined grains (without pizza), mozzarella cheese, and olive oil ^1^. Stratified analysis according to the disease severity (RF- and ACPA-negative, RF- and/or ACPA-positive). Italy 2018–2019.

		RF- and/or ACPA-Positive				RF- and ACPA-Negative				
Logistic Regression										
Food Items/Groups	Tertile Categories	Number of Subjects in Remission/ Active RA	OR	95% CI		Number of Subjects in Remission/ Active RA	OR	95% CI		p_hetero_^3^
**DAS28-CRP**										
Pizza	II (0.065; 0.142]	58/28	0.435	0.211	0.897	43/31	2.397	0.854	6.733	
	III (0.142; 1]	9/3	0.195	0.039	0.969	5/0	NE	NE	NE	0.001
Refined grains (without pizza)	II (0.142; 2.357]	45/30	1.741	0.757	4.006	21/17	1.767	0.543	5.745	
	III (2.357; 9.675]	42/26	1.115	0.437	2.846	34/16	1.319	0.360	4.829	0.788
Mozzarella cheese	II (0.065; 0.142]	57/33	0.682	0.328	1.420	36/25	2.018	0.729	5.581	
	III (0.142; 1]	25/12	0.369	0.141	0.968	21/6	0.931	0.241	3.603	0.234
Olive oil ^2^	II (1; 3]	67/32	0.537	0.269	1.071	45/28	1.216	0.449	3.288	0.114
**SDAI**										
Pizza	II (0.065; 0.142]	33/53	0.204	0.087	0.478	19/55	1.808	0.579	5.645	
	III (0.142; 1]	4/8	0.161	0.032	0.825	3/2	0.333	0.021	5.228	0.020
Refined grains (without pizza)	II (0.142; 2.357]	21/54	0.814	0.326	2.033	11/27	0.479	0.117	1.965	
	III (2.357; 9.675]	21/47	0.546	0.200	1.492	18/32	0.350	0.076	1.616	0.838
Mozzarella cheese	II (0.065; 0.142]	27/63	0.576	0.242	1.370	13/48	1.968	0.643	6.026	
	III (0.142; 1]	15/22	0.200	0.068	0.586	12/15	0.645	0.168	2.470	0.283
Olive oil ^2^	II (1; 3]	33/66	0.567	0.267	1.202	16/57	4.075	1.263	13.147	0.008
**Robust linear regression**										
**Food items/groups**	**Tertile** **categories**	**Number of** **subjects**	**Beta**	**SE**	***p*** **-value**	**Number of subjects**	**Beta**	**SE**	***p*** **-value**	**p_hetero_^3^**
**DAS28-CRP**										
Pizza	II (0.065; 0.142]	86	−0.584	0.163	<0.001	74	0.196	0.178	0.271	
	III (0.142; 1]	12	−0.675	0.341	0.052	5	−0.379	0.424	0.358	0.001
Refined grains (without pizza)	II (0.142; 2.357]	75	0.349	0.200	0.086	38	−0.117	0.211	0.580	
	III (2.357; 9.675]	68	0.017	0.223	0.941	50	−0.159	0.233	0.499	0.970
Mozzarella cheese	II (0.065; 0.142]	90	−0.261	0.181	0.154	61	0.235	0.179	0.192	
	III (0.142; 1]	37	−0.573	0.230	0.013	27	0.020	0.229	0.931	0.164
Olive oil ^2^	II (1; 3]	99	−0.371	0.161	0.023	73	0.114	0.181	0.527	0.126
**SDAI**										
Pizza	II (0.065; 0.142]	86	−3.232	0.980	0.001	74	1.243	1.130	0.271	
	III (0.142; 1]	12	−5.279	2.053	0.012	5	−1.877	2.688	0.471	<0.001
Refined grains (without pizza)	II (0.142; 2.357]	75	1.342	1.118	0.240	38	−0.733	1.324	0.584	
	III (2.357; 9.675]	68	0.184	1.246	0.883	50	−1.970	1.465	0.185	0.941
Mozzarella cheese	II (0.065; 0.142]	90	−0.475	1.041	0.657	61	1.762	1.133	0.122	
	III (0.142; 1]	37	−2.439	1.320	0.063	27	0.704	1.444	0.628	<0.001
Olive oil ^2^	II (1; 3]	99	−2.094	0.962	0.034	73	−0.267	1.146	0.817	<0.001

^1^ Estimated from multiple logistic regression models, or robust linear regression models adjusted for age (≤55, >55 years old), sex, education (maximum level attained: primary school, middle school, high school, university), total energy intake, body mass index (BMI, <18.5, 18.5–24, 25–29, ≥30 kg/m^2^), alcohol-drinking intensity (never a drinker, <1, 1–<2, ≥2 drinks/day), cigarette-smoking status (never, former, current), presence of any therapy (yes, no), conventional synthetic (cs)DMARDs (disease-modifying anti-rheumatic drugs, no, yes), biologic (b)DMARDs (no, yes), targeted synthetic (ts)DMARDs (no, yes), steroids (no, yes), and disease duration (≤5, 5–≤10, 10–≤15, 15–≤25, >25 years). The reference category, I, includes the lowest consumers of each food item/group, i.e., participants consuming up to the first tertile value included. ^2^ For the olive oil food item, the third tertile category (III) was not available, because the second tertile value was equal to 3, which was also the maximum consumption attainable for olive oil. ^3^ *p*-value for the heterogeneity of effect estimates across strata. Frequency of consumption of each food item/group according to the reference portion (see below): Never = 0, 1/month = 0.032, 2/month = 0.065, 1/week = 0.142, 2–3/week = 0.357, 4–5/week = 0.643, 1/day = 1, 2–3/day = 2.5, 4–5/day = 4.5. Reference portion: **Pizza**: half a pizza; **Refined grains**: Bread: 100 g, Pasta or Rice: 80 g; Breakfast cereals: 30 g, and Corn: 100 g; **Mozzarella cheese**: 1 serving of mozzarella; **Olive oil**: 1 tablespoon. Abbreviations: ACPA, anti-citrullinated protein antibodies; CI, confidence interval; DAS28-CRP, Disease Activity Score based on 28 joints with C-reactive protein; NE, not estimable; OR, odds ratio; RF, rheumatoid factor; SDAI, Simplified Disease Activity Index; SE, standard error.

**Table 4:** The odds ratios of rheumatoid arthritis disease activity, and the corresponding 95% confidence intervals (upper panel) and beta coefficients representing an increase/decrease in the mean DAS28-CRP or SDAI and the corresponding standard errors (lower panel), according to the highest tertile categories of consumption of the four investigated food items/groups: pizza, refined grains (without pizza), mozzarella cheese, and olive oil ^1^. Stratified analysis according to the disease duration (≤15 years, >15 years). Italy 2018–2019.

		Disease Duration >15 years				Disease Duration ≤15 years				
Logistic Regression										
Food Items/Groups	Tertile Categories	Number of Subjects in Remission/ Active RA	OR	95%CI		Number of Subjects in Remission/ Active RA	OR	95%CI		p_hetero_^3^
**DAS28-CRP**										
Pizza	II (0.065; 0.142]	39/25	0.55	0.236	1.28	62/34	0.971	0.454	2.081	
	III (0.142; 1]	8/0	NE	NE	NE	6/3	1.057	0.186	5.995	0.026
Refined grains (without pizza)	II (0.142; 2.357]	28/20	0.880	0.337	2.294	38/27	5.994	2.130	16.866	
	III (2.357; 9.675]	27/20	0.791	0.271	2.308	49/22	1.718	0.532	5.550	0.064
Mozzarella cheese	II (0.065; 0.142]	38/26	0.310	0.124	0.779	55/32	1.707	0.757	3.852	
	III (0.142; 1]	19/7	0.178	0.049	0.650	27/11	1.109	0.388	3.171	0.011
Olive oil ^2^	II (1; 3]	47/31	0.481	0.210	1.104	65/29	0.794	0.376	1.678	0.394
**SDAI**										
Pizza	II (0.065; 0.142]	20/44	0.389	0.138	1.092	32/64	0.563	0.261	1.215	
	III (0.142; 1]	3/5	0.190	0.020	1.830	4/5	0.378	0.060	2.373	0.835
Refined grains (without pizza)	II (0.142; 2.357]	15/33	0.308	0.091	1.036	17/48	1.091	0.415	2.867	
	III (2.357; 9.675]	10/37	0.591	0.151	2.320	29/42	0.540	0.195	1.498	0.055
Mozzarella cheese	II (0.065; 0.142]	15/49	0.513	0.169	1.563	25/62	0.990	0.441	2.222	
	III (0.142; 1]	9/17	0.299	0.074	1.207	18/20	0.406	0.153	1.080	0.733
Olive oil ^2^	II (1; 3]	20/58	0.615	0.231	1.634	29/65	1.155	0.559	2.387	0.457
**Robust linear regression**										
**Food items/groups**	**Tertile categories**	**Number of subjects**	**Beta**	**SE**	***p*** **-value**	**Number of subjects**	**Beta**	**SE**	***p*** **-value**	**p_hetero_^3^**
**DAS28-CRP**										
Pizza	II (0.065; 0.142]	64	−0.517	0.209	0.015	96	−0.174	0.142	0.221	
	III (0.142; 1]	8	−1.120	0.479	0.018	9	−0.062	0.323	0.855	0.016
Refined grains (without pizza)	II (0.142; 2.357]	48	0.179	0.247	0.474	65	0.349	0.177	0.053	
	III (2.357; 9.675]	47	−0.041	0.278	0.884	71	0.081	0.198	0.685	0.822
Mozzarella cheese	II (0.065; 0.142]	64	−0.402	0.218	0.074	87	0.134	0.144	0.353	
	III (0.142; 1]	26	−0.651	0.285	0.023	38	−0.094	0.184	0.606	0.038
Olive oil ^2^	II (1; 3]	78	−0.384	0.201	0.059	94	−0.094	0.136	0.494	0.585
**SDAI**										
Pizza	II (0.065; 0.142]	64	−2.760	1.291	0.035	96	−1.167	0.814	0.152	
	III (0.142; 1]	8	−5.174	2.963	0.073	9	−2.356	1.855	0.235	<0.001
Refined grains (without pizza)	II (0.142; 2.357]	48	−0.251	1.434	0.864	65	2.043	1.056	0.058	
	III (2.357; 9.675]	47	−0.575	1.614	0.723	71	0.282	1.178	0.813	<0.001
Mozzarella cheese	II (0.065; 0.142]	64	−1.611	1.296	0.226	87	1.243	0.851	0.148	
	III (0.142; 1]	26	−2.699	1.695	0.110	38	−0.471	1.088	0.667	<0.001
Olive oil ^2^	II (1; 3]	78	−2.193	1.202	0.073	94	−0.196	0.781	0.806	<0.001

^1^ Estimated from multiple logistic regression models or robust linear regression models adjusted for age (≤55, >55 years old), sex, education (maximum level attained: primary school, middle school, high school, university), total energy intake, body mass index (BMI, <18.5, 18.5–24, 25–29, ≥30 kg/m^2^), alcohol drinking intensity (never drinker, <1, 1–<2, ≥2 drinks/day), cigarette smoking status (never, former, current), presence of any therapy (yes, no), conventional synthetic (cs)DMARDs (disease-modifying anti-rheumatic drugs, no, yes), biologic (b)DMARDs (no, yes), targeted synthetic (ts)DMARDs (no, yes), steroids (no, yes), rheumatoid factor (RF) (negative, positive), and anti-citrullinated protein antibodies (ACPA) (negative, positive). The reference category, I, includes the lowest consumers of each food item/group, i.e., participants consuming up to the first tertile value included. ^2^ For the olive oil food item, the third tertile category (III) was not available, because the second tertile value was equal to 3, which was also the maximum consumption attainable for olive oil. ^3^ *p*-value for the heterogeneity of effect estimates across strata. Frequency of consumption of each food item/group according to the reference portion (see below): Never = 0, 1/month = 0.032, 2/month = 0.065, 1/week = 0.142, 2–3/week = 0.357, 4–5/week = 0.643, 1/day = 1, 2–3/day = 2.5, 4–5/day = 4.5. Reference portion: **Pizza**: half a pizza; **Refined grains**: Bread: 100 g, Pasta or Rice: 80 g; Breakfast cereals: 30 g, and Corn: 100 g; **Mozzarella cheese**: 1 serving of mozzarella; **Olive oil**: 1 tablespoon. Abbreviations: CI, confidence interval; DAS28-CRP, Disease Activity Score based on 28 joints with C-reactive protein; NE, not estimable; OR, odds ratio; SDAI, Simplified Disease Activity Index; SE, standard error.

## Data Availability

The data presented in this paper are available upon request from the corresponding authors.

## Supplementary Materials

The following are available online at https://www.mdpi.com/article/10.3390/nu15153449/s1, Table S1: Distribution of 365 rheumatoid arthritis patients according to selected characteristics. Italy 2018–2019.
