# Supplementary material for: Does Pizza Consumption Favor an Improved Disease Activity in Rheumatoid Arthritis?
Source: Nutrients. 2023 Aug 4;15(15):3449. doi: 10.3390/nu15153449 (PMC10421216; doi:10.3390/nu15153449)
Supplement: Supplementary file 1 [file nutrients-15-03449-s001.zip › nutrients-2530632-supplementary.pdf]

nutrients

MDPI

Article

Does pizza consumption favor an improved disease activity in rheumatoid arthritis?

Roberta De Vito, Maria Parpinel, Michela Carola Speciani, Federica Fiori, Rachele Bianco, Roberto Caporali, Francesca Ingegnoli, Isabella Scotti, Tommaso Schioppo, Tania Ubiali, Maurizio Cutolo, Giuseppe Grosso, Monica Ferraroni, and Valeria Edefonti

Supplementary Results

The sensitivity analyses targeted study subjects with either normal blood pressure or not reporting to suffer from gastritis or gastro-esophageal reflux. The derived point estimates generally aligned with those from the corresponding models in the complete-case analysis. The major differences were observed in logistic regression models assessing the effect of dietary habits on SDAI-based disease activity. Specifically, in both sensitivity analyses, the OR for pizza did not reach significance anymore for third vs. first tertile categories of consumption (OR: 0.282, 95% CI: 0.061-1.292 and OR: 0.585, 95% CI: 0.116-2.939 for no gastritis/gastro-esophageal reflux and for normal blood pressure, respectively). The same happened for mozzarella cheese when normal blood pressure participants were considered (OR: 0.463, 95% CI: 0.170-1.263, for III vs. I). On the other hand, the OR of RA activity, as measured by DAS28-CRP, became significant for olive oil: the OR was equal to 0.536 (95% CI: 0.294-0.977), when subjects reported neither gastritis nor gastro-esophageal reflux, vs. an OR of 0.729 (95% CI: 0.443-1.201) in the complete-case analysis, for subjects consuming >1 tablespoon/day (II) vs. ≤1 tablespoon/day of olive oil (I). In the stratified analyses, the wider CIs generally observed included 1 for pizza and mozzarella cheese when DAS28-CRP was considered in participants showing normal blood pressure and RF and/or ACPA positivity. The corresponding estimates were: OR=0.214 (95% CI: 0.042–1.088) vs. OR=0.195 (95% CI: 0.039–0.969) in the complete-case stratum analysis for pizza and OR=0.414 (95% CI: 0.156–1.096) vs. OR=0.369 (95% CI: 0.141–0.968) in the complete-case stratified analysis for mozzarella cheese, when comparing third vs. first tertile categories of consumption. On the contrary, within the same sensitivity analysis and stratum, participants consuming >1 tablespoon/day (II) vs. ≤1 tablespoon/day (I) of olive oil had significantly lower risk of active RA according to DAS28-CRP: OR=0.466 (95% CI: 0.230–0.944) vs. OR=0.537 (95% CI: 0.269–1.071) in the complete-case stratum.

Table S1. Distribution of 365 rheumatoid arthritis patients according to selected characteristics. Italy 2018-2019.

Characteristics	
**Age at baseline**[1], years, median (IQR)	58.46 (47.81-69.03)
≤55 years, N (%)	155 (42.47)
>55, N (%)	210 (57.53)
**Female**, N (%)	287 (78.63)
**Education**[2]	
Primary school, N (%)	49 (13.42)
Middle school, N (%)	79 (21.64)
High school, N (%)	134 (36.71)
University, N (%)	87 (23.84)
**Body Mass Index**, Kg/m^2, median (IQR)	23.63 (21.00-26.78)
<18.5, N (%)	22 (6.03)
18.5 - 25, N (%)	207 (56.71)
25 - 30, N (%)	94 (25.75)
≥30, N (%)	42 (11.51)
**Cigarette smoking status**[2]	
Never, N (%)	189 (51.78)
Former, N (%)	118 (32.33)
Current, N (%)	55 (15.07)
**Alcohol drinking intensity**	
Never, N (%)	106 (29.04)
<1 drink/day, N (%)	194 (53.15)
1 - 2 drinks/day, N (%)	29 (7.95)
≥2 drinks/day, N (%)	36 (9.86)
**Disease duration**, years, median (IQR)	12.81 (8.08-20.72)
≤5, N (%)	51 (13.97)
5 - 10, N (%)	84 (23.01)
10 - 15, N (%)	76 (20.82)
15 - 25, N (%)	89 (24.38)
>25, N (%)	65 (17.81)
Positivity for **rheumatoid factor**, N (%)	196 (53.70)
Positivity for **anti-citrullinated protein antibodies**, N (%)	186 (50.96)
**DAS28-CRP**, median (IQR)	2.21 (1.61-3.02)
Remission, N (%)	227 (62.19)
Low activity, N (%)	60 (16.44)
Moderate activity, N (%)	64 (17.53)
High activity, N (%)	14 (3.84)
**SDAI**, median (IQR)	6.30 (3.01-11.81)
Remission, N (%)	108 (29.59)
Low activity, N (%)	155 (42.47)
Moderate activity, N (%)	81 (22.19)

High activity, N (%)	21 (5.75)
**Swollen joint count** (0-28), median (IQR)	0 (0-1)
**Tender joint count** (0-28), median (IQR)	0 (0-2)
**C-reactive protein**, mg/dL, median (IQR)	2 (0.6-5.57)
**General Health** (0-100), median (IQR)	70 (60-85)
**Physician's Global Assessment** (0-10), median (IQR)	2 (0-4)
**Comorbidities**	
Chronic renal failure, N (%)	4 (1.1)
Arterial hypertension, N (%)	122 (33.42)
Coronary artery disease, N (%)	19 (5.21)
Diabetes mellitus, N (%)	19 (5.21)
Gastro-esophageal reflux disease, N (%)	70 (19.18)
Inflammatory bowel disease, N (%)	2 (0.55)
Gastritis, N (%)	32 (8.77)
Esophagitis, N (%)	5 (1.37)
**Presence of any therapy**	
Yes, N (%)	30 (8)
No, N (%)	335 (91)
**Conventional Synthetic (cs)DMARDs**	
Yes, N (%)	118 (34.2)
No, N (%)	247 (65.8)
**Biologic (b)DMARDs**	
Yes, N (%)	187 (51.2)
No, N (%)	178 (48.8)
**Targeted Synthetic (ts)DMARDs**	
Yes, N (%)	7 (1.9)
No, N (%)	358 (98.1)
**Steroids**	
Yes, N (%)	209 (57.3)
No, N (%)	156 (42.7)

[1] Age was calculated as the difference between date of interview and date of birth.
[2] The sum does not add to the total because of a few missing values in education (16 subjects, 4.38%) and cigarette smoking status (3 subjects, 0.82%).
Abbreviations: DAS28-CRP: Disease Activity Score based on 28 joints – C-reactive protein; DMARDS: Disease Modifying Anti-Rheumatic Drugs; IQR: Interquartile range; SDAI: Simplified Disease Activity Index.

Disclaimer/Publisher's Note: The statements, opinions and data contained in all publications are solely those of the individual author(s) and contributor(s) and not of MDPI and/or the editor(s). MDPI and/or the editor(s) disclaim responsibility for any injury to people or property resulting from any ideas, methods, instructions or products referred to in the content.
